# Postprandial serum endotoxin in healthy humans is modulated by dietary fat in a randomized, controlled, cross-over study

**DOI:** 10.1186/s12944-016-0357-6

**Published:** 2016-11-05

**Authors:** Joshua M. Lyte, Nicholas K. Gabler, James H. Hollis

**Affiliations:** 1 Department of Food Science and Human Nutrition, Iowa State University, Ames, IA 50011 USA; 2 Department of Animal Science, Iowa State University, Ames, IA 50011 USA; 3 Alimentary Pharmabiotic Centre Microbiome Institute, University College Cork, Cork, T12 YT20 Ireland

**Keywords:** Endotoxin, Lipopolysaccharide, Diet, Lipid, Fat, Oil, Inflammation

## Abstract

**Background:**

High-fat diets may contribute to metabolic disease via postprandial changes in serum endotoxin and inflammation. It is unclear how dietary fat composition may alter these parameters. We hypothesized that a meal rich in n-3 (ω3) fatty acids would reduce endotoxemia and associated inflammation but a saturated or n-6 (ω6) fatty acid-rich meal would increase postprandial serum endotoxin concentrations and systemic inflammation in healthy adults.

**Methods:**

Healthy adults (*n* = 20; mean age 25 ± 3.2 S.D. years) were enrolled in this single-blind, randomized, cross-over study. Participants were randomized to treatment and reported to the laboratory, after an overnight fast, on four occasions separated by at least one week. Participants were blinded to treatment meal and consumed one of four isoenergetic meals that provided: 1) 20 % fat (control; olive oil) or 35 % fat provided from 2) n-3 (ω3) (DHA = 500 mg; fish oil); 3) n-6 (ω6) (7.4 g; grapeseed oil) or 4) saturated fat (16 g; coconut oil). Baseline and postprandial blood samples were collected. Primary outcome was defined as the effect of treatment meal on postprandial endotoxemia. Serum was analyzed for metabolites, inflammatory markers, and endotoxin. Data from all 20 participants were analyzed using repeated-measures ANCOVA.

**Results:**

Participant serum endotoxin concentration was increased during the postprandial period after the consumption of the saturated fat meal but decreased after the n-3 meal (*p* < 0.05). The n-6 meal did not effect a different outcome in participant postprandial serum endotoxin concentration from that of the control meal (*p* > 0.05). There was no treatment meal effect on participant postprandial serum biomarkers of inflammation. Postprandial serum triacylglycerols were significantly elevated following the n-6 meal compared to the n-3 meal. Non-esterified fatty acids were significantly increased after consumption of the saturated fat meal compared to other treatment meals.

**Conclusions:**

Meal fatty acid composition modulates postprandial serum endotoxin concentration in healthy adults. However, postprandial endotoxin was not associated with systemic inflammation in vivo.

**Trial registration:**

This study was retrospectively registered at clinicaltrials.gov as NCT02521779 on July 28, 2015.

## Background

In recent years, accumulating research has demonstrated a link between dietary fat and endogenous endotoxin in relation to metabolic inflammation [1, 2]. Current evidence suggests that dietary fat modulates plasma endotoxin concentration which is associated with low-grade systemic inflammation and is implicated in the development of dysregulated metabolism [3–6]. Endotoxin, also known synonymously as lipopolysaccharide (LPS), is considered a major predisposing factor for inflammation-associated diseases such as atherosclerosis, sepsis, obesity, type 2 diabetes and Alzheimer’s [7–9]. However, the effect of dietary fat on the plasma endotoxin remains poorly understood as it is not known if it is the total fat content of a meal or its fatty acid composition exerts the primary effect.

Previous studies have shown that consuming a high fat meal (50 g fat) is associated with a post-prandial increase in plasma [4] and serum [10] endotoxin concentrations in adult humans. Limited evidence also suggests that the type of fatty acid may be important with one study showing that consuming oleic acid-rich peanuts reduced serum endotoxin in healthy men compared to when they consumed standard peanuts [11]. A recent study reported that, using an ex vivo swine intestine model, mucosal to serosal endotoxin transport was decreased with a high docosahexaenoic acid (DHA) and eicosapentaenoic acid (EPA)-meal but increased after a saturated fatty acid rich (coconut oil)-meal; however, the inflammatory response in these animals was not investigated [12].

High-fat diets have been linked with elevated blood concentrations of multiple inflammatory markers including interleukin (IL)-6 [13], IL-8 [14], and tumor necrosis factor-α (TNF- α) [15]. As bacterial endotoxin is recognized by the body’s innate immune system and is a potent initiator of inflammation processes [16], a postprandial increase in circulating endotoxin offers a potential mechanistic explanation for the inflammatory response [4]. However, while blood endotoxin concentration has been associated with incidence of inflammation [17] and metabolic dysfunction [18, 19], others have reported an in vitro*,* but not in vivo*,* postprandial relationship between blood endotoxin concentration and inflammation following the consumption of high-fat meals [4].

Because the link between dietary fat intake, endotoxin, and inflammation is unclear, the primary objective of this study was to determine the effect of dietary fatty acid composition on postprandial endotoxemia in healthy subjects. Based on a previous study [12] we hypothesized that postprandial endotoxin concentrations and markers of inflammation in healthy adults would be increased by meals high in saturated or n-6 fatty acids, but reduced by meals enriched in n-3 polyunsaturated fatty acids. Pre- and postprandial serum was assayed for endotoxin, inflammatory markers, and metabolites.

## Methods

### Human subjects

Male and female participants (Table 1) [(average age 25 y (SD: 3.2 y); average body mass index (BMI) 22.4 kg/m^2^ (SD: 2 kg/m^2^); average weight 65.6 kg (SD: 8 kg)] were recruited via a mass email to faculty, staff, and students of Iowa State University or through personal contact during Spring and Fall of 2014. Inclusion criteria were age between 18 and 40 y, BMI ≥ 19.9 and ≤ 24.9, less than 2 kg weight change in the previous 3 months and a willingness to eat the test meals. Exclusion criteria were the presence of acute or chronic disease, use of tobacco products, consumption of more than 21 units of alcohol per week, use of anti-inflammatory medication, or a history of macronutrient malabsorption.

**Table 1:** Demographics of subjects (*n* = 20) that successfully completed the study^a^

	Male	Female
Number of subjects	12	8
Average age (y)	25	25
Weight (kg)	68.9	59.4
Body mass index	22.7	22.3

^a^ Participant information about race and ethnicity was not collected

### Study design

**Fig. 1:**
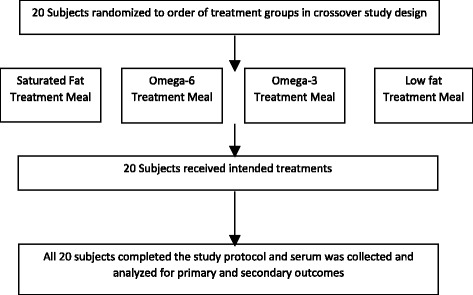
Flow chart of subjects (*n* = 20) through the study

This study used a randomized, single-blind, cross-over design. After being recruited to the study, participants (*n* = 20) were randomized to a treatment order (Fig. 1). Participants were required to report to the laboratory first thing in the morning following an overnight fast of at least 12 hours on four separate occasions each separated by at least 7 days.

On the evening before each test session, participants were required to eat a standardized meal that provided 50 % of the participant’s estimated energy requirements and contained 50 % carbohydrate, 15 % protein, and 35 % fat. The participant’s estimated daily energy requirements were determined using validated equations [20]. On the morning of each test session, participants were required to arrive at the laboratory at 0715. An indwelling catheter was inserted into the antecubital vein of each participant’s non-dominant arm and a baseline blood draw (10 mL) was taken (time point t = 0). Participants were then provided with the relevant test meal that was consumed in its entirety within 15 min. On each occasion, participants were blinded to which treatment meal they would be provided that morning. All treatment meals were served in a uniform, visually indistinguishable fashion to avoid participant recognition of treatment meal. Participants remained in the laboratory for five and a half hours during which additional blood draws (10 mL per blood draw) were taken at time points t0+ 1, 2, 3, 4, and 5 h. During this time, participants were allowed to perform sedentary activities (e.g. watch television, use their computer) but were not allowed to consume any food or drink except water. Following the final blood draw at t0+ 5 h, the indwelling catheter was removed and the participant permitted to leave the laboratory.

Immediately following venipuncture, blood was allowed to clot in pyrogen-free blood collection tubes for 45 min, followed immediately by centrifugation (15 min, 2000 × *g*, 4 °C), and stored in pyrogen-free tubes (Fisher Scientific, Pittsburgh, PA) at −80 °C until further analysis or transferred into standard plastic screw-cap vials (Quest Diagnostics, Madison, NJ) according to company instructions for same-day pickup and shipment to Quest Diagnostics for analysis of serum metabolites.

### Test meals

Each test meal provided 25 % of the participant’s estimated daily energy requirements as determined using validated equations [20]. The test meal was a porridge made with quick-ready oatmeal (Hy-Vee Supermarkets, Ames, IA) prepared with water according to the manufacturer’s instructions. To this oatmeal, 1) coconut oil (Spectrum, Lake Success, NY), 2) olive oil (Crisco, Orville, OH), 3) grapeseed oil (Pompeian, Baltimore, MD) or 4) fish oil (Carlson, Arlington Heights, IL) was added in order to provide each of the four test meals a unique macronutrient (Table 2) and fatty acid profile (Table 3). 60 g ± 17 g of hard-boiled egg (Crystal Farms 6 peeled hard-boiled eggs ready-to-eat, Lake Mills, WI), 201 g ± 26 g of fat-free skim milk (HyVee fat free skim milk, Des Moines, IA), and 184 g ± 33 g of orange juice (Tropicana Pure Premium No Pulp, Bradenton, FL) were served with the porridge. The participants were required to eat the test meal in its entirety within 15 min of serving.

**Table 2:** Treatment meal composition

	Low-fat		High fat (n-3)		High fat (n-6)		High fat (saturated)	
Carbohydrate (%)^a^	65		50		50		50	
Protein (%)	15		15		15		15	
Total fat (%)	20		35		35		35	
Saturated fat (%)	5		10		10		15	
Total n-6 fatty acids (%)	2		2		7		2	
EPA + DHA fatty acids (%)	0		0.5		0		0	
	Mean	SEM	Mean	SEM	Mean	SEM	Mean	SEM
Energy (kcal/g)^b^	4.96^a^	0.04	4.90^a^	0.01	5.09^a^	0.05	5.22^b^	0.01
Endotoxin (EU/g)^b^	89.66^a^	3.29	72.97^b^	1.07	72.72^b^	0.63	65.98^c^	0.51

^a^ Values (%) are based on 25 % of participant estimated daily energy requirements

^b^ Each test meal was bombed in duplicate as described in Methods. Test meals were assayed in duplicate using LAL kinetic chromogenic endotoxin assay. Values within the same row but with different superscript letters are significant at the *p* < 0.05 level

**Table 3:** Fatty acid composition of test meals^a, b^

Lipid (C:D)	Lipid common name	Low-fat	High fat (n3)	High fat (n6)	High fat (saturated)
8:0	Caprylic	-	1.1	-	2.9
10:0	Capric	-	0.9	1.1	2.7
12:0	Lauric	-	8.5^c^	8.4^c^	23.8^d^
14:0	Myristic	0.2^c^	5.0^d^	3.8^d^	10.6^e^
16:0	Palmitic	16.4	17.1	14.2	15.9
16:1	Palmitoleic	1.1	2.5	0.7	0.8
18:0	Stearic	4.3	4.9	5.0	4.7
18:1 n9 cis	Oleic	57.3^b^	35.6^c^	31.4^c^	24.8^e^
18:2 n6 cis	Linoleic	16.8^b^	13.0^d^	31.2^e^	10.3^d^
20:0	Arachidic	0.2	0.1	0.1	0.1
18:3 n3	α-Linolenic	0.8	0.7	0.5	0.4
20:1 n9	Eicosenoic	0.3	0.4	0.2	0.2
20:4 n6	Arachidonic	0.6	0.8	0.6	0.7
20:5 n3	Eicosapentaenoic	-	3.0	-	-
22:6 n3	Docosahexaenoic	-	2.3	-	-
Other		2.0	4.1	2.8	2.1
n-6:n-3		21.7	2.3	63.6	27.5
Saturated		21.1	32.6	32.6	60.7
n-3		0.8	6	0.5	0.4
n-6		17.4	13.8	31.8	11

^a^All values are expressed as percent of total fatty acids from a lipid extract prepared from duplicate samples of each test meal as described in the Methods; values within the same row but with different superscript letters (e.g. c, d, or e) are significantly different at the *p* < 0.05 level

^b^ -; not detected

### Bomb calorimetry

The energy (kcal/g) of each test meal was determined using bomb calorimetry. Each test meal was prepared identical as if it were to be served to a participant. Meals were homogenized using a food-grade commercial blender (Model HBH450, Hamilton Beach Commercial, Glen Allen, VA) on high setting for 1 min and then passed through a 2 mm sieve (Advantech 2.00 mm USA standard testing sieve No. 10, New Berlin, WI). Aliquots were weighed and then lyophilized at −55 °C using a Uni-Trap Model 10–100 (The Virtis Company, Gardiner, NY). Lyophilized samples were ground and passed through a 1 mm screen before being pelleted into duplicate ~1.00 g pellets using a manual pellet press (Parr Instrument Co., Moline, IL). Pellets were placed within a model 1108 oxygen bomb (Parr Instrument Co.) and bombed in a 6200 Isoperibol calorimeter (Parr Instrument Co.). Each test meal was analyzed in duplicate with an accepted CV of less than 2 % between duplicate samples. Calorimetry standard 1.00 g benzoic acid pellets (Parr Instrument Co.) were used to calibrate each bomb before the analysis of test meal pellets.

### Limulus amebocyte lysate assays

Participant serum and test meal endotoxin concentrations were determined using the kinetic chromogenic limulus amebocyte lysate (LAL) assay (Lonza, Switzerland). Endotoxin concentration was expressed as endotoxin units (EU) per mL. Meals were freshly-prepared identically as if they were to be served to a participant. Prepared meals were then placed into separate sterile Whirl-pak filter bags (Nasco, Fort Atkinson, WI) and homogenized in a Stomacher 3500 (Seward, Davie, FL) for 2 min [21, 22]. Aliquots of the homogenized meal filtrate were collected into pyrogen-free tubes and stored at −80 °C until analysis by the endotoxin assay. To minimize any impact of repeated freeze-thaw cycle on endotoxin-activity, serum and meal samples were thawed a single time for endotoxin assay. A positive product control (PPC) recovery test of an endotoxin spike of known concentration was performed in serum and in homogenized meal filtrate per LAL kit manufacturer instructions (data not shown). A dilution ratio of homogenized meal filtrate or serum: LAL-grade water of 1:100 provided a PPC recovery of the endotoxin spike within manufacturer recommendation of 50-200 %. Meticulous attention was given to the handling of serum and meal samples and the materials used in the endotoxin analysis to avoid contamination with exogenous endotoxin. Pipet tips, dilution tubes, 96-well microtiter plates, and reagent reservoir for use with a multi-channel pipet were all certified to contain endotoxin concentrations < 0.005 EU/mL (Lonza, Switzerland).

Participant serum and meal samples were diluted 1:100 in LAL-grade water (Lonza, Switzerland) and heated at 70 °C for 15 min in order to heat-inactivate enzymatic activity that may affect endotoxin detection by the LAL method. 100 μL of each heat-treated serum and meal sample was plated in duplicate on endotoxin-free 96-well plates and incubated in a PowerWave HT microplate reader (Biotek, Winooski, VT) at 37 °C for 10 min. At the completion of the incubation period, the plate was removed from the plate reader and 100 μL of LAL reagent was added to each sample well on the 96-well plate. The plate was then read at an absorbance of 405 nm according to the LAL-assay manufacturer’s instructions. The assay result of each serum sample was accepted if the intra-assay CV between duplicate wells was below 10 %. Endotoxin concentrations were generated based on a standard curve constructed from a kit-supplied endotoxin standard prepared in LAL-grade water. The standard curve was constructed according to manufacturer’s instructions and provided an endotoxin detection range from 0.005 EU/mL – 50 EU/mL. All LAL-kits utilized in this study were verified as from the same manufacturing lot in order to eliminate potential impact on endotoxin measurements due to inter-lot kit variation.

### Biomarkers of inflammation assays

A magnetic, fluorescent bead-based immunoassay (Bio-Plex pro human cytokine group I 4-plex kit, Bio-Rad, Hercules, CA) was used to assay all serum samples for the cytokines IL-6, IL-8, IL-10, and TNF-α. All Bio-Plex assays were performed on a Bio-Plex 200 system (Bio-Rad) and run according to manufacturer instructions. In brief, serum samples were diluted 1:4 in kit-supplied sample diluent and run in duplicate on kit-supplied 96-well plates. A standard curve for each analyte was constructed from the kit-supplied lyophilized standard reconstituted in kit-provided standard diluent and a minimum bead count of 50 beads per analyte was acquired for each well. Bio-Plex Manager software standard edition version 6.1 (Bio-Rad) was used to collect the mean fluorescent intensity of each cytokine-specific bead region and convert the data using the standard curve into reportable concentrations (pg/mL). The reported concentration of each analyte was the average between duplicate wells.

C-reactive protein (CRP) content of each serum sample was determined via a nephelometric method performed by Quest Diagnostics (test ordering code 4420). CRP results were reported via delivered mail and expressed in mg/dL.

### Metabolite assays

All serum samples were assayed via spectrophotometric methods for concentrations of triacylglycerols (test ordering code 896) and non-esterified fatty acids (NEFA) (test ordering code 449) by Quest Diagnostics. Triacylglycerols are expressed in mg/dL, while NEFA are expressed as mmol/L.

### Fatty acid analysis

Freshly-prepared porridge test meals were homogenized with egg, skim milk, and orange juice in a food-grade commercial blender (Model HBH450, Hamilton Beach Commercial, Glen Allen, VA) on high setting for 1 min. Homogenized test meals were then passed through a 2.00 mm sieve (Advantech 2.00 mm USA standard testing sieve No. 10, New Berlin, WI) and aliquots were collected for immediate lipid extraction according to established methodology [23]. Extracted lipids were dried under nitrogen gas before undergoing transesterification into fatty acid methyl esters using an acetyl chloride/methanol method [24]. In brief, 1 mL of methanol was added to 40 mg of dried extracted lipids. Samples were then vortexed, during which 100 μL of acetyl chloride was added, and immediately purged under nitrogen gas and capped. Samples were heated for 1 h at 80 °C, and then allowed to cool to room temperature. Esterified lipids were transferred to a gas chromatography vial, purged under nitrogen gas and stored at −20 °C until injection into the gas chromatograph (GC). Unless otherwise noted, all chemical reagents were analytical standard-grade and purchased from Acros Organics (Bridgewater, NJ).

Fatty acid methyl esters were analyzed in duplicate on a GC (Model 3800; Varian Analytical Instruments, Walnut Creek, CA) equipped with a SP-2380 (100 m × 0.25 mm I.D., 0.20 μm) capillary column (Supelco, Bellefonte, PA). Helium was the carrier gas with a flow rate of 2 mL/min. The GC injection port temperature was 220 °C and operated in standard split/splitless mode. The GC oven was maintained at 70 °C for 4 min, then increased to 175 °C at a rate of 13 °C/min and isothermally held for 27 min, and then increased to a final temperature of 215 °C at a rate of 4 °C/min and isothermally held for 28 min. Test meal fatty acid profiles were analyzed using commercial software (Varian Star Chromatography Workstation Version 6.41, Walnut Creek, CA). Peak identification was validated by relative retention times with known reference standards (Supelco, Bellefonte, PA) and methyl tricosanoate (Nu-Chek Prep, Elysian, MN).

### Statistical analysis

The primary outcome of this study was the effect of test meal fatty acid composition on postprandial serum endotoxin concentration. The secondary outcome was the effect of the test meal on the serum postprandial concentration of biomarkers of inflammation and metabolites. Mean and standard error were calculated for all study variables. Treatment effects of the test meal on serum endotoxin, inflammatory markers and metabolites were analyzed using a mixed model ANCOVA using treatment and time point as repeated measures and baseline as a covariate. All post-hoc, pairwise comparisons were performed using Bonferroni adjustments. Measured endotoxin values were log-transformed to normalize data distribution before parametric statistical analysis. Shapiro-Wilk normality test was employed to confirm log-transformed data were Gaussian. Data was analyzed using SPSS software (version 22; IBM, Armonk, NY). Statistical significance was set at *p* < 0.05. A power calculation was done for Bonferroni multiple comparisons and it was estimated that a sample size of 16 individuals would be sufficient to detect a one SD difference with α = 0.05 and β = .80. Participants were randomly assigned to treatment sequence using a Latin Square design.

## Results

### Test meal fatty acid profiles

The unique fatty acid compositions of each of the four test meals are presented in Table 3. The saturated fat test meal contained a high percentage (60.7 %) of several saturated fatty acids found in coconut oil including lauric, myristic, and palmitic fatty acids. Compared to the saturated fat meal, the n-3 test meal contained a lower total percentage of saturated fatty acids (32.6 %). Moreover, the n-3 test meal contained both docosahexaenoic and eicosapentaenoic omega-3 fatty acids that were not present in the saturated fat meal. The low-fat test meal primarily contained fatty acids found in olive oil, including oleic (57.3 %), palmitic (16.4 %), and linoleic (16.8 %) acids. The n-6 test meal contained a high percentage of fatty acids found in grapeseed oil including linoleic (31.2 %), oleic (31.4 %), palmitic (14.2 %), and stearic (5.0 %) acids.

### Test meal energy and endotoxin contents

The energy content (kcal/g) and endotoxin (EU/g) contents of each of the four test meals is reported in Table 2. The mean energy content of the four test meals was 5.0 ± 0.04 kcal/g (dry weight basis). The meal endotoxin concentrations ranged from 65.9 to 89.6 EU/g (Table 2).

### Kinetic chromogenic LAL assay of serum endotoxin

All subjects, regardless of treatment group or day of laboratory visit, had detectable levels of endotoxin in their serum at baseline measurement. Average participant baseline endotoxin concentration was 0.365 ± 0.09 EU/mL. To ensure baseline endotoxin values were not the result of exogenous contamination from the materials used in the isolation of serum, control vessels were included and assayed identical to the vessels that contained human tissue. All control tubes had endotoxin levels of <0.005 EU/mL as measured by the LAL assay. Accuracy of baseline endotoxin values was validated by repeat LAL assay of randomly selected baseline serum samples on two separate days via two separate LAL kits with the same manufacture lot number (data not shown).

There was a significant main effect of test meal on serum endotoxin concentration (F(3, 83) = 3.104; *p* < 0.05). Post hoc analysis revealed that serum endotoxin was lower following the n-3 meal compared to the saturated fat meal (*p* < 0.05). Comparison of the mean endotoxin values of the three high fat diets to the low fat diet did not yield a statistically significant (*p* < 0.05) difference suggesting that absolute fat intake does not influence post-prandial endotoxemia. In addition, there was a significant main effect of time on serum endotoxin (F(4, 229) = 2.972; *p* < 0.05). Post hoc analysis revealed a statistically significant difference between timepoints 60 min and 240 min and 120 min and 240 min (*p* < 0.05). The treatment x time interaction was not statistically significant.

### Quantitative determination of multiple serum biomarkers of systemic inflammation

Participants’ baseline and postprandial serum concentrations of C-reactive protein measured below the detectable limit (<0.10 mg/dL) of the assay performed by Quest Diagnostics. Fluorescent bead-based immunoassay (Bio-Plex) of each participant serum sample revealed concentrations of IL-6, IL-8, IL-10, and tumor necrosis factor-α were not significantly (*p* > 0.05) different from baseline measurement or between treatment meals (data not shown).

### Measurement of serum triacylglycerol and non-esterified fatty acids

**Fig. 2:**
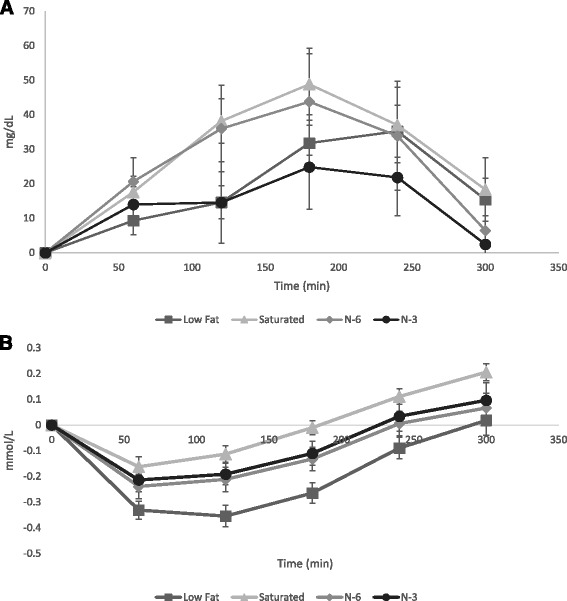
Change from baseline effect of treatment meals on postprandial serum metabolite concentrations (mean ± SEM). **a** Triacylglycerol (mg/dL). **b** Non-esterified fatty acids (mmol/L). Participant serum triacylglycerol and non-esterified fatty acids were analyzed via spectrophotometric methods. Repeated measures ANCOVA with baseline as a covariate was performed with posthoc analysis as described in Methods. Serum triacylglycerols were significantly elevated (*p* < 0.05) following the n-6 fatty acid rich test meal when compared to the n-3 fatty acid rich meal. Serum non-esterified fatty acids were significantly higher (*p* < 0.05) in the saturated fatty acid rich meal compared to the other test meals

Mean postprandial serum concentrations of triacylglycerols and non-esterified fatty acids displayed similar change-from-baseline trends irrespective of test meal (Fig. 2). The mean participant baseline measurement of serum triacylglycerols concentration (mg/dL) was 89 ± 2. There was a significant main effect of treatment meal on serum triacylglycerols concentration (F(3, 58) = 3.865; *p* < 0.05). Post-hoc analysis indicated serum triacylglycerols concentration following the n-6 meal to be statistically higher (*p* < 0.05) than after consumption of the n-3 meal (Fig. 2a). A significant main effect was also found of time on triacylglycerols concentration (F(4, 255) = 29.805; *p* < 0.05). Post hoc analysis revealed a statistically significant difference between time points 60 min and 120 min, 60 min and 180 min, 60 and 240 min, 120 min and 180 min, 120 min and 300 min, 180 min and 300 min, 240 min and 300 min. The treatment x time interaction was not statistically significant.

Mean participant baseline concentration of NEFA (mmol/L) was 0.407 ± 0.019. A significant main effect was were found for test meal (F(3, 105) = 8.859; *p* < 0.05) on serum NEFA concentration. A significant main effect was also found for time (F(4, 256) = 179.456; *p* < 0.05) on NEFA serum concentration (Fig. 2b). Following the saturated fat test meal NEFA serum concentration was significantly different (*p* < 0.05) than had the participant consumed any of the other test meals. Post hoc analysis revealed a statistically significant difference between time points 60 min and 180 min, 60 min and 240 min, 60 min and 300 min, 120 min and 180 min, 120 min and 240 min, 120 min and 300 min, 180 min and 240 min, 240 min and 300 min. Treatment meal x time point did not reach statistical significance.

## Discussion

The results of this study demonstrate that the modulatory role of dietary fat intake on postprandial endogenous endotoxin concentration in healthy adult men and women is influenced by dietary fatty acid composition, but not the fat content of a meal. Although serum endotoxin was found to increase after a saturated fatty acid rich meal or decrease after an n-3 PUFA enriched meal, markers of in vivo inflammation were unaffected. Whether an elevated blood endotoxin concentration following a single high-fat meal may lead to inflammation in people is unclear as other studies have reported an association [4, 10] while others did not find evidence linking postprandial endotoxin with inflammation [25]. This inconsistently-described relationship may be, in part, explained because previous studies involving humans have not fully explored the role of dietary fat composition in mediating outcomes of postprandial endotoxin and inflammation. The present study therefore, to the best of our knowledge, is one of the initial studies to directly examine this relationship in humans.

A clearly observed trend was generated from the effect over time of test meal on subject postprandial serum endotoxin concentration (Table 4). The low-fat meal, which provided 20 % of its energy from fat, was included in this study in order to examine whether a meal’s percent energy from fat influenced postprandial endotoxin concentration. Notably, the higher-fat test meals, in which each meal provided 35 % of calories from fat, were not different from the low-fat meal in their effect on postprandial serum endotoxin (data not shown). This suggests that in an isoenergetic series of meals, a higher percentage of fat does not differentially alter postprandial serum endotoxin concentration.

**Table 4:** Effect of treatment meal on participant postprandial serum endotoxin concentration over time^a^

Meal	Time postprandial (minutes)					
	0	60	120	180	240	300
Low Fat	0.39 ± 0.09	0.45 ± 0.14	0.37 ± 0.10	0.28 ± 0.03	0.25 ± 0.02	0.32 ± 0.07
Saturated	0.27 ± 0.03	0.39 ± 0.11	0.51 ± 0.14	0.49 ± 0.17	0.31 ± 0.08	0.38 ± 0.20
N-3	0.43 ± 0.15	0.29 ± 0.07	0.26 ± 0.03	0.24 ± 0.02	0.26 ± 0.02	0.26 ± 0.02
N-6	0.36 ± 0.12	0.59 ± 0.20	0.46 ± 0.16	0.36 ± 0.09	0.24 ± 0.02	0.31 ± 0.09

^**a**^ All values expressed as mean ± SEM. Measured endotoxin expressed as endotoxin units/mL; Participant serum (*n* = 20 samples/time-point) endotoxin was determined in duplicate using the kinetic chromogenic LAL endotoxin assay. Treatment effect was analyzed following the log-transformation of measured endotoxin values using repeated measures ANCOVA with baseline as a covariate as described in Methods. The n-3 fatty acid rich test meal effected a significantly lower (*p* < 0.05) postprandial serum endotoxin than the saturated fatty acid rich test meal

Among the higher-fat test meals, we found that the fatty acid composition of the meal had a significant effect on postprandial outcome serum endotoxin (Table 4). In particular, the saturated fat and n-3 fat meals elicited opposite effects on postprandial serum endotoxin. A similar relationship between saturated and n-3 fats on serum endotoxin has been previously demonstrated to occur in swine [12]. However, in mice, a diet rich in saturated fat had no significant effect on postprandial endotoxin [26]. The reasons why conflicting results would be obtained from different species is not entirely clear. The comparative similarity of study design between the swine study and the present study, which yielded similar results, included the selection of coconut oil as the saturated fat treatment, and fish oil as the n-3 fat treatment. Laugerette et al. [26] recently reported dissimilar findings when using milk-fat or palm, sunflower, and rape seed oils as dietary treatments. Specifically, saturated fat-rich palm oil resulted in the lowest blood endotoxin concentration whereas rapeseed oil, low in saturated fat, resulted in the highest blood endotoxin concentration. Another reason for conflicting results is that the measurement of serum endotoxin in swine and our present study followed ingestion of a single treatment meal, compared to mice that were fed for 8-weeks on differing dietary treatments and then endotoxemia assessed. Anatomical differences between species may also contribute to the different results obtained in each species [27, 28]. For example, mouse intestinal tract physiology is less similar to human than is that of swine [29–32].

That the n-6 meal raised postprandial endotoxin concentration but was not significantly different from other higher-fat treatments agrees with previous findings in swine where n-6-rich vegetable oil was not significantly different in treatment effect on serum endotoxin than n-3 or saturated fat meals [12]. The postprandial trend in our data for the effect of the n-6 meal where serum endotoxin appeared higher than that of the n-3 treatment but lower than the saturated fat-treatment, mirrors the treatment effect trend found in swine [12].

The intestinal epithelial interface is known to play a role in immune recognition between host and non-host [33]. Toll-like receptor (TLR)-4 is one of the innate immune receptors involved in the recognition of the lipid A antigenic portion of endotoxin [34]. Lipid A often contains saturated fatty acids common in human diets [34–36]. While saturated fatty acids and n-3 PUFA have been demonstrated to reciprocally modulate human TLR-4 [34, 35], saturated non-esterified fatty acids (NEFA) in the blood of healthy Malaysian adults due to dietary fat intake did not increase inflammatory markers [36]. Likewise, in our study, n-3 and saturated fat test meals were found to effect significantly different serum NEFA but not inflammatory marker outcomes.

Test meal fat composition and postprandial changes in serum endotoxin did not associate with in vivo inflammation in any of our serum samples. While serum endotoxin concentration did uniquely change during the postprandial phase of each test meal, none of the test meals of our study caused a significant rise in endotoxin concentration when compared to baseline measurement (Table 4). One possibility for this observation was that the subjects were healthy, young, and fed only a single meal to assess postprandial endotoxin. Although a separate study reported a rise in plasma IL-6 at 120 min postprandial in healthy men after a single meal containing 33 g fat [37], we did not observe an associated peak of IL-6. Similar to our findings, others have reported no association between serum endotoxin and IL-6 in healthy men [5]. An intake of approximately 24 oz of orange juice was reported to blunt the pro-inflammatory response and rise in circulating endotoxin following a high-fat meal [38]. As the present study included less than 8 oz of orange juice as part of each test meal, it is unlikely that orange juice contributed a significant anti-inflammatory effect.

The occurrence of inflammation following feeding does not appear to be dependent on changes in blood endotoxin concentrations. While in the present study we did not find a significant rise in endotoxin concentration following the feeding of any of the treatment meals, other studies that found postprandial increases in blood endotoxin after consumption of a single high-fat meal, likewise, did not report changes in inflammation. In male smokers, plasma CRP concentration, an acute phase protein, was not found to increase following a high-fat meal despite a significant rise in postprandial endotoxin when compared to baseline measurement [4]. Morbidly obese men that had measurable serum CRP before ingestion of a 50 g fat meal, but following consumption had no increase in CRP despite a significant rise in serum endotoxin [10]. The absence of an increase in inflammation, including no detectable production of CRP, in our samples may be, in part, due to the exclusion criteria in selecting for volunteers for the present study. Undetectable concentrations of TNF-α in human plasma have been previously reported to coincide with measurable increases in plasma endotoxin after a single high-fat meal that contained 50 g of butter [4]. Although endotoxin may induce in vitro IL-8 [39] and IL-10 [40] expression in human endothelial and Kupffer cells, respectively, we found no in vivo evidence of this relationship in our samples. Likewise, a separate group also reported no association between in vivo measurement of IL-8 and the feeding of a high-fat meal in humans [25]. This may suggest that human cell line production of certain pro- and anti-inflammatory cytokines after in vitro challenge by exogenous endotoxin does not adequately mimic in vivo conditions after dietary fat intake.

## Conclusions

In conclusion, our results demonstrate that the composition of dietary fat, but not a meal’s percent calories from fat, is significant in determining postprandial changes in blood endotoxin concentration in healthy adults in vivo. Although endotoxemia has been associated with low-grade inflammation, we did not observe an increase or decrease in postprandial endotoxemia to modulate inflammation. As such, we could not prove a link between postprandial endotoxemia and inflammation following the consumption of a single meal unique in dietary fatty acid composition. Postprandial endotoxemia was found to be increased following consumption of a meal rich in saturated fatty acids but decreased after a meal containing n-3 polyunsaturated fatty acids. Future studies should investigate whether long-term repeated consumption of such meals would chronically influence blood endotoxin and presence of inflammation.

## Abbreviations

CRP: C-reactive protein
DHA: Docosahexaenoic acid
EPA: Eicosapentaenoic acid
EU: Endotoxin unit
GC: Gas chromatograph
LAL: Limulus amebocyte lysate
NEFA: Non-esterified fatty acids
TNF-α: Tumor necrosis factor-α
